# Factors Affecting the Extent of Patients’ Electronic Medical Record Use: An Empirical Study Focusing on System and Patient Characteristics

**DOI:** 10.2196/30637

**Published:** 2021-10-28

**Authors:** Lavlin Agrawal, Theophile Ndabu, Pavankumar Mulgund, Raj Sharman

**Affiliations:** 1 The State University of New York at Buffalo, School of Management Buffalo, NY United States

**Keywords:** electronic medical record, patient safety, caregiver, chronic conditions, HINTS dataset, patient technology acceptance model

## Abstract

**Background:**

Patients’ access to and use of electronic medical records (EMRs) places greater information in their hands, which helps them better comanage their health, leading to better clinical outcomes. Despite numerous benefits that promote health and well-being, patients’ acceptance and use of EMRs remains low. We study the impact of predictors that affect the use of EMR by patients to understand better the underlying causal factors for the lower use of EMR.

**Objective:**

This study aims to examine the critical system (eg, performance expectancy and effort expectancy) and patient characteristics (eg, health condition, issue involvement, preventive health behaviors, and caregiving status) that influence the extent of patients’ EMR use.

**Methods:**

We used secondary data collected by Health Information National Trends Survey 5 cycle 3 and performed survey data analysis using structural equation modeling technique to test our hypotheses. Structural equation modeling is a technique commonly used to measure and analyze the relationships of observed and latent variables. We also addressed common method bias to understand if there was any systematic effect on the observed correlation between the measures for the predictor and predicted variables.

**Results:**

The statistically significant drivers of the extent of EMR use were performance expectancy (β=.253; *P*<.001), perceived behavior control (β=.236; *P*<.001), health knowledge (β=–.071; *P*=.007), caregiving status (β=.059; *P*=.013), issue involvement (β=.356; *P*<.001), chronic conditions (β=.071; *P*=.016), and preventive health behavior (β=.076; *P*=.005). The model accounted for 32.9% of the variance in the extent of EMR use.

**Conclusions:**

The study found that health characteristics, such as chronic conditions and patient disposition (eg, preventive health behavior and issue involvement), directly affect the extent of EMR use. The study also revealed that issue involvement mediates the impact of preventive health behaviors and the presence of chronic conditions on the extent of patients’ EMR use.

## Introduction

### Background

An electronic medical record (EMR), also called the online medical record system, is a kind of software that stores clinical information such as medication lists, laboratory results, physician observations, immunizations, allergies, and discharge information [1]. Due to the impetus provided by the Health Information Technology for Economic and Clinical Health Act, EMR usage by providers and hospital administrators surged significantly, leading to improved documentation, data availability, and streamlined order entry to decrease prescription errors [2].

Although physician adoption and use of EMRs have been widely investigated [3-10], patients’ use of EMR warrants further research. Patient adoption and use of EMRs represent a different phenomenon contrasted with physician adoption and use of EMR. For example, patients are not subject to organizational pressures prevalent in physician adoption and use decisions. Other factors differentiating the two contexts derive from the fact that patients may not be familiar with the technology to access EMR, and their understanding of clinical terms may be limited.

It is crucial to increase patient use of EMR for various consequential reasons, specifically patient empowerment. According to the World Health Organization, patient empowerment is a process through which people gain greater control over decisions and actions affecting their health [11]. Patients’ access to and use of medical records empowers them to take a more active role in managing their health [12] by placing more information in their hands, which can improve clinical outcomes. Further, a patient portal built on top of EMR offers several benefits to patients, including continuous monitoring of health information, improved interactions with providers, better patient engagement in health management, scheduling appointments, and messaging physicians [13].

This research investigates the factors that influence patients’ adoption and use of EMRs using an extended version of the patient technology acceptance model (PTAM). Specifically, we focus on the effect of salient patient characteristics such as health conditions, issue involvement, preventive health behaviors, and caregiving status on the adoption and use of EMR systems because they remain understudied.

### Hypotheses and Proposed Model

#### Overview

From a theoretical perspective, the unified theory of acceptance and use of technology has been employed to understand technology adoption and use in general [14]. It was adapted to the health care context with the addition of patient-centered factors (psychomotor, visual, and cognitive aspects) to study patients’ adoption of technology [15] and was called PTAM. It considers perceived usefulness, perceived ease of use, perceived behavior control, subjective norm, and patient characteristics (psychomotor, visual, and cognitive aspects) as main predictors of the adoption and use of health information technologies by patients. PTAM was originally developed by Or et al [15] for the general context of patient adoption of technology. Since EMR is a specific technology for storing medical records, we had to adapt it to our context.

**Figure 1:**
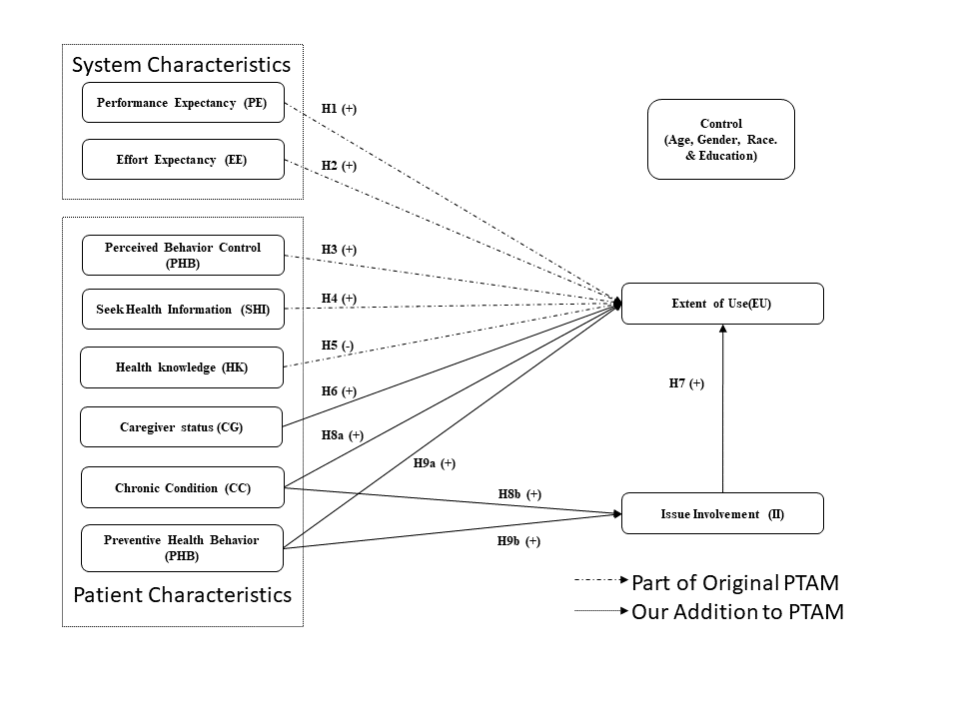
Research model. PTAM: patient technology acceptance model

In this study, we not only considered factors such as performance expectancy (perceived usefulness), effort expectancy (perceived ease of use), perceived behavior control, but also extended the model with patient characteristics that are not part of the original PTAM, such as health condition, preventive health behavior, issue involvement, and patients’ caregiving status. Following that, we introduced issue involvement as a mediator between the extent of EMR use and (1) chronic conditions and (2) preventive health behavior. Age, gender, education, and race were used as control variables in our study. Our proposed research model is illustrated in Figure 1. This adaptation contributes to the development of a theoretical foundation that could be used to improve our understanding of patients’ EMR use.

#### Performance Expectancy

Davis [16] defined perceived usefulness as one of the key predictors of new system adoption. Venkatesh et al [14] extended this notion of perceived usefulness by defining performance expectancy as the degree to which a person feels that using a system will help them perform a job more efficiently. In keeping with this understanding, we refer to performance expectancy as the degree to which the patient believes that using EMRs helps them monitor their health. Venkatesh et al [14] theorized that performance expectancy drives the intention to use information systems. Several researchers have also identified performance expectancy as one of the critical predictors of eHealth acceptance and use [15,17-21]. Because EMRs improve patient engagement and empower patients to access their health information anytime and anywhere [22], we propose the following hypothesis:

- H1: Performance expectancy is positively related to the extent of EMR use.

#### Effort Expectancy

Extending the ease of use construct [16], Venkatesh et al [14] defined effort expectancy as the degree of comfort associated with system use. Consistent with Venkatesh et al [14], we define effort expectancy as the degree of ease associated with understanding the health information in the online medical record. Venkatesh et al [14] suggested that effort expectancy has a positive effect on use intentions. Many researchers have also identified effort expectancy as one of the critical predictors of health adoption and use [15,19-21]. Studies have confirmed that ease of use is an essential predictor of intended use. Therefore, we propose the following hypothesis:

- H2: Effort expectancy is positively related to the extent of EMR use.

#### Perceived Behavioral Control

Or et al [15] defined perceived behavioral control as an individual’s perception of their ability to do something (ie, self-efficacy). Many researchers have suggested that self-efficacy directly determines intent to use, especially online and mobile applications [23]. Lack of self-efficacy with computers and the internet is one of the most frequently identified barriers to adopting and using patient portals [24,25]. Turner et al [26] confirmed that the lack of comfort with computers is one of the common barriers to patient adoption of a portal. Thus, we posit that competency with technology is more likely to generate confidence in using EMRs. We hypothesize the following:

- H3: Perceived behavioral control is positively related to the extent of EMR use.

#### Seek Health Information

Seek health information (SHI) refers to individuals’ urge to look for health-related information. Wilson and Lankton [27] argued that individuals seeking health information are more likely to adopt eHealth applications because such applications increase the availability of health information and reduce the effort needed to access that information. Or et al [15] extended the same concept and theorized that individuals who need to review health information are more likely to accept and use technology. As EMRs can help individuals get their health information and health history, and based on prior studies regarding health-information-seeking behavior, we propose the following hypothesis:

- H4: Seeking health information is positively related to the extent of EMR use.

#### Health Knowledge

Or et al [15] defined health knowledge as the knowledge that individuals feel they have about their health condition. Fowles et al [28] reported that sharing medical records with individuals has a modest positive impact on their knowledge. Wilson and Lankton [27] stated that an individual with little knowledge about their health is more likely to accept the eHealth tools managed by providers. Therefore, we hypothesize the following:

- H5: Health knowledge is negatively related to the extent of EMR use.

#### Caregiving

Caregiving implies providing paid or unpaid support and making medical decisions for a patient when appropriate [29,30]. In this study, we consider only an unpaid caregiver (generally family members or friends) who is currently caring for or making health care decisions for someone with a medical condition, behavioral or physical disability, or other condition.

King et al [31] provided evidence that caregivers use assistive health technologies (ie, any product, hardware, or software used to increase, maintain, or improve the functional capabilities of individuals with disabilities) to better care for children with a physical disability. Studies have also suggested that caregiver status strongly influences portal use, especially for caregivers who provide care for patients with chronic health conditions [32,33]. Caregivers’ exposure to EMRs enhances their proficiency in using EMRs and makes them more likely to use EMRs themselves. Thus, we propose the following hypothesis:

- H6: Caregiving individuals are more likely to use EMRs extensively.

#### Issue Involvement

Issue involvement refers to how personally relevant people find an issue [34]. Abdelhamid et al [35] define issue involvement in the health care domain as “how relevant a specific health issue is to a patient.” A more involved patient frequently visits providers, has several diseases, or has a severe health condition [36]. Consistent with Angst and Agarwal [36], we consider a patient with more physician visits (measured in our study as the number of physician visits in the past 12 months) as more involved with issues. Prior studies have demonstrated a positive relationship between issue involvement and the use of eHealth products [35,36]. Ross et al [37] argued that issue involvement has a significant positive impact on the adoption and use of EMRs. They found that EMRs better prepare patients for their upcoming visits with physicians by enhancing their knowledge of their medical condition, increasing their sense of control, and allowing them to seek clarification about treatment. Accordingly, we hypothesize the following:

- H7: Issue involvement is positively related to the extent of EMR use.

#### Chronic Conditions

Wagner et al [38] and Kruse et al [39] advocated for patients’ use of health care systems and available resources to self-manage their health, especially for chronic health conditions. Studies have also suggested that patients with chronic conditions are more likely to use available eHealth applications to be better informed and manage their health [40-42]. A literature review [18] confirmed that patients with comorbidities are more likely to use electronic personal health record systems. Therefore, we posit that EMRs help patients track their improvement or deterioration in health and make informed decisions to better take care of themselves. Hence, we hypothesize that patients with existing chronic conditions are more likely to use EMRs.

Broemeling et al [43] demonstrated that a person with a chronic condition is more likely to visit a physician regularly. We, therefore, hypothesize that chronic conditions affect issue involvement (ie, frequency of physician visits). A higher number of chronic conditions may motivate patients to check their conditions, diagnostics, or prescriptions more closely, leading to greater EMR use. Those patients may also want to ensure that their records are correct to improve patient safety. Hence, we hypothesize that the extent of the chronic condition increases issue involvement and the need for frequent doctor visits.

- H8a: The presence of chronic conditions is positively related to the extent of EMR use.
- H8b: The presence of chronic conditions is positively related to issue involvement.

#### Preventive Health Behavior

Kasl et al [44] defined preventive health behavior as “any activity undertaken by a person who believes himself to be healthy for preventing disease or detecting disease in an asymptomatic stage.” People with such drive are likely to monitor their health conditions through their EMRs. Studies have suggested that individuals use the available resources and skills to engage in preventive health behavior [35,45]. These resources may include accessing their records in EMR systems and seeking the help of physicians.

In psychology, motivation is described as a reason that drives action [46]. Thus, we posit that health motivators—in this case, preventive health behavior—influence people to engage in behaviors that improve their health outcomes and encourage frequent EMR use. This reasoning also finds resonance with earlier studies on the benefits of EMRs and the quality of health outcomes [47-49]. In this study, we consider a person to be involved in preventive health behavior if they exercise and eat fruits and vegetables as recommended by the US Centers for Disease Control and Prevention (CDC). This understanding is similar to the operationalization by Hart et al [50].

Näslund [51] concluded that an individual engaging in preventive health behavior would have more doctor visits; this tendency is more pronounced in women. Grembowski et al [52] argued that individuals with preventive health behavior are more likely to initiate preventive care and early treatment. Other studies have suggested that health information technology plays a significant role in self-management [53,54]. Therefore, we hypothesize that individuals practicing preventive health behavior are more likely to visit their physicians often and use EMRs.

- H9a: Preventive health behavior is positively related to the extent of EMR use.
- H9b: Preventive health behavior is positively related to issue involvement.

Figure 1 and Table 1 summarize the hypothesis and definitions of the variables used in this model.

**Table 1:** Summary of hypothesis and variables.

Hypothesis		Variable	Defined in this study as	Relates
**DV^a^** **: extent of EMR^b^** **use (EU)^c^**				
	H1	Performance expectancy (PE)	Degree to which the patient believes that using EMRs help them monitor their health	Positively
	H2	Effort expectancy (EE)	Degree of ease associated with understanding the health information in the online medical record	Positively
	H3	Perceived behavioral control (PBC)	Individual's perception of their ability to use electronic means	Positively
	H4	Seek health information (SHI)	Whether an individual looked for information about health or medical topic from any source	Positively
	H5	Health knowledge (HK)	If an individual is confident about ability to take good care of health	Negatively
	H6	Caregiving status (CG)	If an individual is providing unpaid care to a patient	Positively
	H7	Issue involvement (II)	Frequency of doctor visits in last 12 months	Positively
	H8a	Chronic conditions (CC)	Number of chronic conditions an individual has	Positively
	H9a	Preventive health behavior (PHB)	An individual is considered to have preventive health behavior if they exercise, eat fruits and vegetables as recommended by CDC^d^	Positively
**DV: Issue involvement (II)^e^**				
	H8b	Chronic conditions (CC)	Number of chronic conditions an individual has	Positively
	H9b	Preventive health behavior (PHB)	An individual is considered to have preventive health behavior if they exercise, eat fruits and vegetables as recommended by CDC	Positively

^a^DV: dependent variable.

^b^EMR: electronic medical record

^c^Number of times the online medical record has been accessed in the last 12 months.

^d^CDC: Centers for Disease Control and Prevention.

^e^Frequency of doctor visits in the last 12 months.

## Methods

### Data Source

We used data collected between January and May of 2019 by the National Cancer Institute (NCI) for Health Information National Trends Survey (HINTS) 5 cycle 3 to test our hypotheses. NCI administered a paper-based questionnaire and an online questionnaire to survey participants with an overall response rate of 30.3%. This survey was completed by 5438 participants. These data are publicly available and can be accessed at the HINTS website [55].

We filtered the data to include only those respondents who had used EMRs at least once during the previous 12 months. The resulting sample size was 2110. Data did not include outliers. Additionally, missing values on critical variables were less than 5%.

### Measurements

The main dependent variable, the extent of EMR by the individual, was measured with a single item. System characteristics variables, performance expectancy and effort expectancy, were also measured with single items. Single items are acceptable if the question does not leave room for interpretation [56] and is used in information systems research that uses structural equation modeling (SEM) in the health care domain [35,36].

The patient characteristic, issue involvement, was measured with a single item. Other patient-related characteristics such as caregiving status, seek health information, health knowledge, and perceived behavioral control were each measured with a binary choice question. There were 6 binary-choice questions for chronic conditions. The number of responses for chronic conditions was summed for analysis.

A formative measure of preventive health behavior was constructed using 3 items: the number of cups of fruit each day, the number of cups of vegetables per day, and the number of days per week with moderate exercise. According to the CDC [57], eating 1½ to 2 cups of fruit per day and 2 to 3 cups of vegetables per day is a healthy eating pattern. The CDC also recommends physical activity at least 2 days per week [58]. Based on these recommendations, we calculated the score for preventive health behavior as the sum of the responses to each item. Gender, age, race, income, and education were used as controls in the model. Please refer to Multimedia Appendix 1 for a detailed questionnaire, scale, and how they were used in this study.

### Statistical Analysis

In this paper, we used SEM to conduct a path analysis. Although SEM is predominantly used to model latent variables, it is also applied to conduct path analysis in a mediation model, and in our study, we have 2 mediating relationships. First, issue involvement mediates the relationship between chronic conditions and the extent of EMR use. Second, issue involvement also mediates the relationship between preventive health behavior and the extent of EMR use. Therefore, we use SEM to test the model similar to prior scholars [59-61]. We used SEM with robust diagonally weighted least squares (DWLS) to test the hypotheses. DWLS is ideal for ordinal outcome variables [62-64]. We ran our model in R (version 4.0.2; R Core Team) using the “lavaan-survey” package.

## Results

### Descriptive Statistics

Table 2 shows the descriptive statistics of the survey respondents. The survey included questions about the extent of participants’ EMR use. Other questions focused on our model variables, including performance expectancy, effort expectancy, perceived behavioral control, seek health information, health knowledge, caregiving, chronic conditions, preventive health behavior, and issue involvement.

**Table 2:** Descriptive statistics.

Characteristics		Sample size, n (%)
Total responses		2110 (100)
**Extent of EMR^a^** **use (EU)**		
	1 to 2 times	896 (42.46)
	3 to 5 times	679 (32.18)
	6 to 9 times	280 (13.27)
	10 or more times	255 (12.09)
**Performance expectancy (PE)**		
	Don't use	126 (5.97)
	Not at all useful	26 (1.23)
	Not very useful	145 (6.87)
	Somewhat useful	831 (39.38)
	Very useful	950 (45.02)
**Effort expectancy (EE)**		
	Very difficult	22 (1.04)
	Somewhat difficult	184 (8.72)
	Somewhat easy	979 (46.4)
	Very easy	883 (41.85)
**Number of chronic conditions (CC)**		
	0	696 (32.99)
	1	678 (32.13)
	2	445 (21.09)
	3	209 (9.91)
	4	68 (3.22)
	5	13 (0.62)
	6	1 (0.05)
**Issue involvement (II)**		
	None	86 (4.08)
	1 time	225 (10.66)
	2 times	390 (18.48)
	3 times	336 (15.92)
	4 times	354 (16.78)
	5-9 times	438 (20.76)
	10 or more times	272 (12.89)
**Caregiver (CG)**		
	Yes	383 (18.15)
	No	1682 (79.72)
**Health Knowledge (HK)**		
	Not confident at all	15 (0.71)
	A little confident	57 (2.7)
	Somewhat confident	435 (20.62)
	Very confident	1030 (48.82)
	Completely confident	552 (26.16)
**Perceived behavioral control (PHB)**		
	Yes	1701 (80.62)
	No	385 (18.25)
**Seek health information (SHI)**		
	Yes	1923 (91.14)
	No	164 (7.77)
**Preventive health behavior (PHB)**		
	0	515 (24.41)
	1	729 (34.55)
	2	537 (25.45)
	3	329 (15.59)
**Gender** ** **		
	Male	815 (38.63)
	Female	1259 (59.67)
**Education** ** **		
	High school or less	231 (10.95)
	More than high school	1843 (87.35)
**Race**		
	White	1596 (75.64)
	Black	249 (11.8)
	Others	148 (7.01)
**Income, USD**		
	Less than $20,000	171 (8.1)
	$20,000 to < $35,000	172 (8.15)
	$35,000 to <$50,000	241 (11.42)
	$50,000 to <$75,000	382 (18.1)
	$75,000 or more	957 (45.36)
**Age (years)**		
	Min	18
	Max	97
	Mean	54.21
	SD	16.14

^a^EMR: electronic medical record.

### Reliability and Validity

Table 3 shows the correlations between all the variables. Correlation coefficients are important as a high correlation among independent variables indicates a potential bias in coefficients due to multicollinearity. In this data set, the highest correlation is 0.41 between perceived expectancy and effort expectancy. None of the correlations were greater than 0.5, and they were within the acceptable threshold of 0.6 [65], so multicollinearity was not a concern in this analysis. Table 3 also provides the means and standard deviations for the principal variables.

**Table 3:** Correlation matrix.

	Mean (SD)	EU	EE	PE	PBC	SHI	HK	CG	II	CC	PHB
Extent of EMR use (EU)	1.97 (1.03)	1.00									
Effort Expectancy (EE)	3.33 (0.67)	0.12	1.00								
Performance Expectancy (PE)	4.22 (1.00)	0.26	0.41	1.00							
Perceived behavioral control (PBC)	0.83 (0.38)	0.24	0.10	0.23	1.00						
Seek Health Information (SHI)	0.93 (0.26)	0.07	–0.02	0.02	0.11	1.00					
Health Knowledge (HK)	3.99 (0.80)	–0.04	0.28	0.13	0.04	–0.01	1.00				
Caregiving Status (CG)	0.19 (0.39)	0.06	0.00	0.02	–0.02	–0.01	–0.01	1.00			
Issue Involvement (II)	3.44 (1.71)	0.37	–0.07	0.02	0.08	0.10	–0.14	–0.01	1.00		
Chronic Conditions (CC)	1.20 (1.13)	0.14	–0.08	–0.03	0.02	0.00	–0.27	–0.01	0.26	1.00	
Preventive Health Behavior (PHB)	1.33 (1.00)	0.06	0.08	0.08	0.05	0.05	0.21	0.02	–0.04	–0.17	1.00

### Variance Inflation Factor

We used variance inflation factor (VIF) statistics to determine if data is suffering from multicollinearity. Multicollinearity refers to the linear relationship between 2 or more predictor variables [66]. VIF indicates the increase in the variance of a regression coefficient as a result of multicollinearity. Table 4 shows the VIF for each variable. The VIFs for all variables were well below 5.0, suggesting that the data did not suffer from multicollinearity [67].

**Table 4:** Variance inflation factors.

Variable	EE^a^	PE^b^	PBC^c^	SHI^d^	HK^e^	CG^f^	II^g^	CC^h^	PHB^i^
VIF^j^	1.29	1.26	1.08	1.03	1.20	1.00	1.10	1.16	1.07

^a^EE: effort expectancy.

^b^PE: performance expectancy.

^c^PBC: perceived behavioral control.

^d^SHI: seek health information.

^e^HK: health knowledge.

^f^CG: caregiving status.

^g^II: issue involvement.

^h^CC: chronic conditions.

^i^PHB: preventive health behavior.

^j^VIF: variance inflation factors.

### Common Method Variance

Because the data were self-reported and collected through a single survey, the data may suffer from common method variance (CMV), which hampers the relationship between the variables [68]. Therefore, we assessed CMV bias using a marker variable technique [69]. A marker variable is a variable that is theoretically unrelated to one or more of the principal variables measured in the study and typically has a low correlation with the central variables.

Table 5 shows the correlation between the principal variables and marker variables. The theoretically unrelated construct “enjoy time in sun” (ETS) was used as a marker variable. The correlation between the marker variable ETS and other principal variables was low, meeting the threshold below 0.1 [69], except seek health information, which had a correlation of –0.11 with ETS. Similar findings were obtained using “morning-night person” as a marker variable (see Multimedia Appendix 1). The low correlation of the marker variable with the variables in the model indicates the absence of CMV.

**Table 5:** Correlation with marker variables.

		EU^a^	EE^b^	PE^c^	PBC^d^	SHI^e^	HK^f^	CG^g^	II^h^	CC^i^	PHB^j^
**With marker variable “enjoy time in sun”**											
	Correlation	–0.04	0.03	0.05	–0.03	–0.11	0.03	0.01	-0.07	–0.06	0.01
	*P* value	.07	.18	.02	.23	<.001	.20	.66	.002	.01	.77
**With marker variable “morning-night person”**											
	Correlation	0.03	–0.04	–0.02	–0.01	0.01	–0.09	0.01	0.05	0.03	–0.09
	*P* value	.27	.09	.33	.62	.57	<.001	.78	.03	.27	<.001

^a^EU: extent of EMR use.

^b^EE: effort expectancy.

^c^PE: performance expectancy.

^d^PBC: perceived behavioral control.

^e^SHI: seek health information.

^f^HK: health knowledge.

^g^CG: caregiving status.

^h^II: issue involvement.

^i^CC: chronic conditions.

^j^PHB: preventive health behavior.

### Data Analysis

#### Overview

Since the NCI administered a paper-based questionnaire and an online questionnaire to survey participants, we regressed the dependent variable “extent of EMR use” on the mode of survey administration. We found that the relationship between the two was not significant, which means that the mode of survey administration did not affect the extent of EMR use.

Further, we ran our model in R using the “lavaan-survey” package. The overall fit statistics (χ^2^=78.461; *P*<.001; comparative fit index=0.784, Tucker–Lewis index=0.982, root mean square error of approximation=0.056, root mean square residual=0.000, and goodness-of-fit statistic=0.935) of the structural model indicated a good model fit [70]. The SEM results are shown in Table 6. Table 7 presents the mediation analysis results for issue involvement with chronic conditions and preventive disease behavior.

**Table 6:** Results of structural equation modeling.

Variables		Standard estimates	CI lower	CI upper	*P* value	Significant
**DV^a^: Issue involvement (II)**						
	Chronic conditions (CC)	0.237	0.163	0.261	<.001	Yes
	Preventive health behavior	0.004	–0.047	0.055	.87	No
**DV: Extent of EMR^b^ use (EU)**						
	Performance expectancy (PE)	0.253	0.219	0.340	<.001	Yes
	Effort expectancy (EE)	0.047	–0.009	0.170	.08	No
	Perceived behavioral control (PBC)	0.236	0.544	0.868	<.001	Yes
	Seek health information (SHI)	0.027	–0.099	0.341	.28	No
	Health knowledge (HK)	–0.073	–0.176	–0.028	.01	Yes
	Caregiving status (CG)	0.060	0.037	0.309	.01	Yes
	Issue involvement (II)	0.353	0.343	0.430	<.001	Yes
	Chronic conditions (CC)	0.071	0.013	0.127	.02	Yes
	Preventive health behavior (PHB)	0.076	0.026	0.145	.01	Yes
	Male	–0.091	–0.321	–0.097	<.001	Yes
	Age	0.055	0.003	0.122	.04	Yes
	High School or More	0.017	–0.134	0.264	.52	No
	Black	0.032	–0.146	0.367	.40	No
	White	0.056	–0.055	0.370	.15	No
	Income	0.010	–0.037	0.054	.71	No

^a^DV: dependent variable.

^b^EMR: electronic medical record.

**Table 7:** Mediation results of structural equation modeling.

Mediation analysis		Standard estimates		CI lower		CI upper		*P* value		Significant
**Chronic condition** **(CC)**										
	Direct	0.071	0.012		0.126		.02		Yes	
	Indirect through Issue Involvement	0.084	0.062		0.103		<.001		Yes	
	Total	0.155	0.099		0.205		<.001		Yes	
**Preventive health behavior (PHB)**										
	Direct	0.076	0.026		0.144		.005		Yes	
	Indirect through Issue Involvement	0.001	–0.018		0.021		.89		No	
	Total	0.077	0.031		0.142		.002		Yes	

#### System Characteristics

H1 theorized a positive relationship between performance expectancy and the extent of EMR use. Our analysis revealed a positive and statistically significant path coefficient between performance expectancy and the extent of EMR use (βPE=.253; *P*<.001; see Table 6). This indicates that higher performance expectancy results in higher EMR use, thus supporting H1. In line with prior research [15,18-20], we theorized a positive relationship between effort expectancy and the extent of EMR use. The path coefficient was positive but statistically nonsignificant (βEE=.047; *P*=0.77; see Table 6). Therefore, additional research is warranted to examine the results further.

#### Patient Characteristics

H3 anticipated a positive relationship between perceived behavioral control and the extent of EMR use. The path coefficient was positive and statistically significant (βPBC=.24; *P*<.001; see Table 6), suggesting that patients’ higher perceived behavioral control results in higher EMR use, supporting H3. This result is consistent with the findings of prior studies relating to perceived behavioral control in other domains.

H4 theorized a positive relationship between SIH and the extent of EMR use. Although the path coefficient was positive, it was statistically nonsignificant (βSHI=.028; *P*=.28; see Table 6). Therefore, H4 was not supported. H5 theorized a negative relationship between health knowledge and the extent of EMR use. The path coefficient was negative and statistically significant (βHK=–.071; *P*=.01; see Table 6). Therefore, H5 was supported. H6 theorized a positive relationship between caregiving status and the extent of EMR use. The path coefficient was positive and statistically significant (βCG=.06; *P*=.01; see Table 6), thus supporting H6.

H7 proposed a positive relationship between issue involvement and the extent of EMR use. The path coefficient was positive and statistically significant (βII=.356, *P*<.001; see Table 6), suggesting that higher issue involvement results in higher EMR use. Thus, H7 was supported. H8a argued that a positive relationship exists between the extent of chronic conditions and the extent of EMR use. The path coefficient was positive and significant (βCC=.071; *P*=.02; see Table 6), suggesting that a patient with more chronic conditions is more likely to use EMRs frequently. Thus, H8a was supported. H8b predicted a positive relationship between the extent of chronic conditions and issue involvement. The path from chronic conditions to issue involvement was positive and statistically significant (βCC=.238; *P*<.001; see Table 6). The mediation analysis suggests that issue involvement partially mediates the effect of chronic conditions on the extent of EMR use.

**Figure 2:**
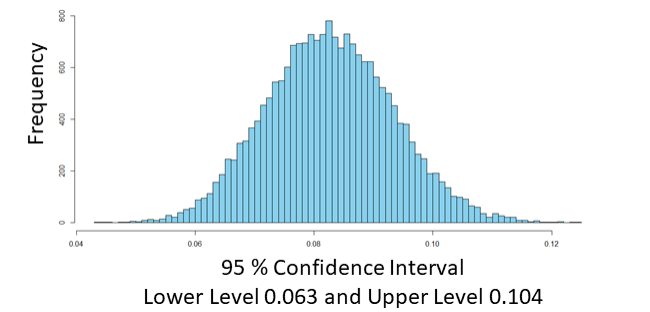
Distribution of indirect effect of chronic conditions on the extent of EMR use. EMR: electronic medical record.

Monte Carlo simulation, also known as the Monte Carlo method or a multiple probability simulation, is a mathematical technique used to estimate the possible outcomes of an uncertain event [71]. We used Monte Carlo simulation to draw a probability distribution of the indirect effect of chronic conditions on the extent of EMR use. Figure 2 provides the probability distribution of the indirect effect of chronic conditions on the extent of EMR use. As the Monte Carlo CI [72] of the indirect effect did not contain zero (CI=0.063-0.104), the mediation of issue involvement between chronic conditions and the extent of EMR use is supported (Table 7). The mediation effect of issue involvement accounted for 48.9% of the impact of chronic conditions on the extent of EMR use.

H9a argued that a positive relationship exists between preventive health behavior and the extent of EMR use. The path coefficient was positive and significant (βPHB=.076; *P*=.005; see Table 6), suggesting that a patient with preventive health behavior is more likely to use EMRs frequently. Thus, H9a was supported. H9b predicted a positive relationship between preventive health behavior and issue involvement. The path coefficient was positive but statistically nonsignificant (βPHB=.001; *P*=.89; see Table 6). Thus, H9b was not supported, which excludes the possibility of any mediation.

**Figure 3:**
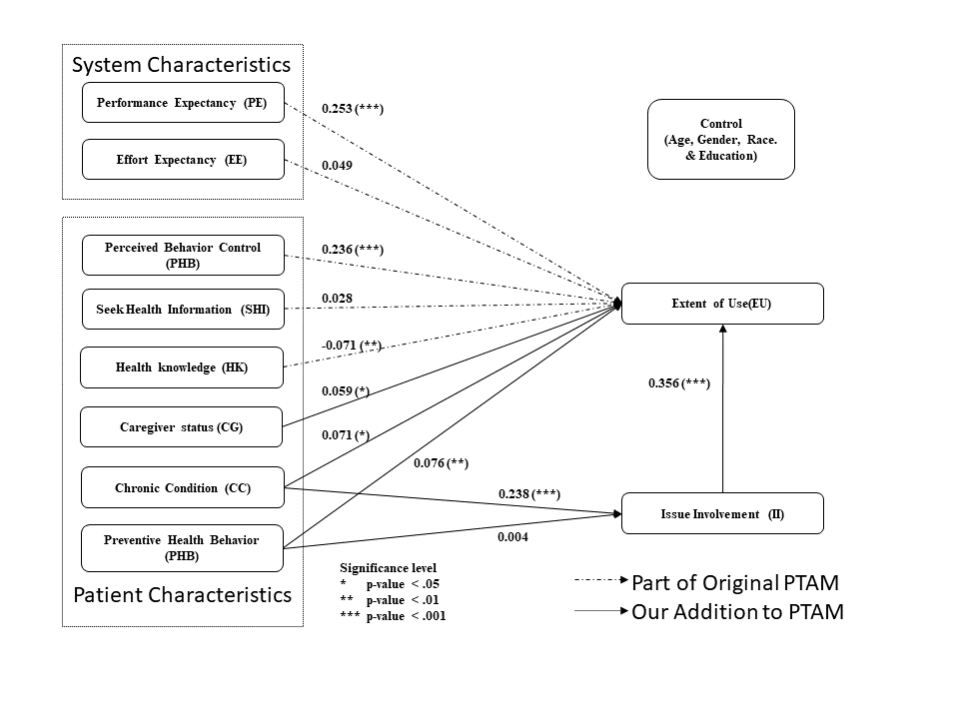
Model results. PTAM: patient technology acceptance model

The study results suggest that, among the patient characteristics, issue involvement (βII=.356; *P*<.001) is the most important factor, followed by perceived behavior control (βPBC=.236; *P*<.001). Figure 3 shows the research model with the path coefficients and their significance, and Table 8 summarizes the study results.

**Table 8:** Summary of study results.

Hypothesis		Independent variable	Result
**DV^a^** **: Extent of EMR^b^ use (EU)**			
	H1	Performance expectancy (PE)	Supported
	H2	Effort expectancy (EE)	Not Supported
	H3	Perceived behavioral control (PBC)	Supported
	H4	Seek health information (SHI)	Not Supported
	H5	Health knowledge (HK)	Supported
	H6	Caregiving status (CG)	Supported
	H7	Issue involvement (II)	Supported
	H8a	Chronic conditions (CC)	Supported
	H9a	Preventive health behavior (PHB)	Supported
**DV: Issue Involvement (II)**			
	H8b	Chronic conditions (CC)	Supported
	H9b	Preventive health behavior (PHB)	Not Supported

^a^DV: dependent variable.

^b^EMR: electronic medical record.

## Discussion

### Theoretical Implications

Our study extends the line of research on the PTAM [15] to explore patients’ use of EMRs and examine the impact of factors that have not been studied by prior research. In line with prior studies [15,18,19], our statistical analysis showed that performance expectancy is a critical system characteristic that influences the patient adoption and use of EMRs. Further, this study finds perceived behavioral control [24-26] and caregiving status [32,33] significant variables for patient adoption and use of EMR. The number of chronic conditions and issue involvement also significantly impact the extent of EMR use by patients.

Our study also finds preventive health behavior to be a significant factor that impacts the extent of EMR use. However, seek health information is not statistically significant. A typical EMR captures the patient’s medical history, including conditions, treatment decisions, medications, procedures, allergies, progress notes, and immunization records [73]. However, as noted by previous studies [15,27], it has not evolved into a source of medical information for patients who usually seek information from providers or the internet. Therefore, it is not surprising that seek health information emerged as an insignificant factor in determining the extent of EMR use. We also find effort expectancy nonsignificant, which is counterintuitive since several studies have found it critical in determining the extent of use [14].

Further, the study results suggest that issue involvement is the most critical patient characteristic, followed by perceived behavioral control as reflected by the model coefficients. Issue involvement refers to an individual’s involvement with their health care issues and reflects their motivation to manage their health-related decisions. Therefore, it is natural that issue involvement emerged as a vital patient characteristic. Perceived behavioral control is another critical determinant of the extent of EMR use.

The study of the effect of chronic conditions and preventive health behaviors on the extent of EMR use is one of the most salient contributions of this article from a theoretical perspective. Unlike others, patients suffering from chronic conditions engage in continuous health monitoring, frequent interactions with medical providers, and an ongoing adjustment of medications. Such patients also require interactions with medical specialists, necessitating the frequent transfer of medical information among several physicians. Given the complexity of care and the patients’ frequent interactions with providers, the statistical significance of chronic conditions as a determinant of EMR use is intuitive. Finally, the study highlights the value of caregiving in sensitizing and educating people about their health. Caregivers witness the challenges patients face and develop an empathetic understanding that increases their awareness and motivates them to adopt better health practices, including keeping track of patients’ health information using EMRs.

In a nutshell, the contributions of this study include insights into how patients’ characteristics and health conditions, along with their perceived system characteristics, influence the extent of EMR use. Our model adds (1) patient characteristics, such as caregiver status and preventive health practices, and (2) health conditions, such as chronic conditions and issue involvement, to the PTAM framework.

### Practical Implications

Understanding the factors that influence the extent of EMR use by patients can be crucial in developing processes and systems that can enhance their adoption and usage. Given the significance of perceived behavioral control, we can institute inventions such as developing high-quality training modules and end-user support services. In addition to demonstrating the product features, training modules can also educate users on the potential value and utility of EMRs, thereby enhancing performance expectancy. The results of this study also suggest that practitioners and providers should dedicate efforts to educating and training patients about the benefits of EMR use. Also, we should promote success stories and best practices of patients using EMRs through case studies. Further, since chronically ill patients are more likely to use EMRs, patient engagement interventions should be directed at them. During the design and development phases, EMRs should also consider the role of caregivers.

### Study Limitations

This study has several limitations. First, the HINTS data relied on self-reported information, so there is potential for CMV [68]. Using the marker variable technique [69], we evaluated that data are not suffering from CMV. Second, the study is based on secondary data and could only use variables present in the data. Certain key variables, such as social norms that may interest a general audience, were not included as these variables were not captured in the survey. Social norms, commonly defined as typical behaviors expected from people, are significant in original PTAM; consequently, the absence of social norms in this study might have inflated some of the estimates. However, since the patient adoption and use of EMRs is a relatively new phenomenon, the social norms around adopting and using EMRs are not well-established. Likely, its impact may not have been significant. Future studies should examine the impact of social factors and analyze their role in the extent of patients’ EMR use. Third, the operationalization of chronic conditions was limited to only 6 major chronic conditions: diabetes, hypertension, heart disease, lung disease, depression, and cancer. To overcome these limitations, researchers should examine factors that affect patients’ EMR use through longitudinal studies that include key variables such as social norms in addition to the variables in the current study.

### Conclusions

Our study contributes to both theory and practice. First, we described how the phenomenon of patient adoption of EMRs is different from physician adoption of EMRs. Second, to understand the factors affecting patients’ EMR use, we adapted the PTAM to the context of EMR use. This resulted in the addition of several new patient characteristics (eg, chronic conditions, preventive health behavior, issue involvement, and caregiving status) that influence the extent of EMR use. Thus, our study contributes to the literature on health information systems. We also found that effort expectancy had no significant effect on the extent of patients’ EMR use. We found that health characteristics, such as chronic conditions, preventive health behaviors, caregiving status, health knowledge, and issue involvement directly affect the extent of EMR use. Our analysis also revealed that issue involvement has a mediating effect on the impact of the extent of the chronic condition on EMR use. EMR enables patients to track their health care history and understand the progress or deterioration in their health conditions. It also provides an opportunity for patients to examine their medical records and get the erroneous medical record corrected. Hence, improving EMR use contributes to patients’ greater control over decisions and actions and adds to the larger goal of patient empowerment.

## Appendix

Multimedia Appendix 1 Operationalization of Constructs (source HINTS 5 cycle 3).

## Abbreviations

CDC: Centers for Disease Control and Prevention
CMV: common method variance
DWLS: diagonally weighted least squares
EMR: electronic medical record
ETS: enjoy time in the sun
HINTS: Health Information National Trends Survey
NCI: National Cancer Institute
PTAM: patient technology acceptance model
SEM: structural equation modeling
VIF: variance inflation factor
