# Supplementary material for: Factors Affecting the Extent of Patients’ Electronic Medical Record Use: An Empirical Study Focusing on System and Patient Characteristics
Source: J Med Internet Res. 2021 Oct 28;23(10):e30637. doi: 10.2196/30637 (PMC8587186; doi:10.2196/30637)
Supplement: Multimedia Appendix 1 [file jmir_v23i10e30637_app1.docx]

## Appendix A: Operationalization of Constructs (source HINTS 5 cycle 3)

| **Construct** | **Question** | **Scale*** | **Coding*** |
| --- | --- | --- | --- |
| Extent of EMR Use (EU) | How many times did you access your online medical record in the last 12 months? | 1: 1 to 2 times  2: 3 to 5 times  3: 6 to 9 times  4: 10 or more times | 1: 1 to 2 times  2: 3 to 5 times  3: 6 to 9 times  4: 10 or more times |
| Performance Expectancy (PE) | In general, how useful is your online medical record for monitoring your health? | 5: don't use  4: Not at all useful  3: Not very useful  2: Somewhat useful  1: Very useful | 1: don't use  2: Not at all useful  3: Not very useful  4: Somewhat useful  5: Very useful |
| Effort Expectancy (EE) | How easy or difficult was it to understand the health information in your online medical record? | 4: Very difficult  3: Somewhat difficult  2: Somewhat easy  1: Very easy | 1: Very difficult  2: Somewhat difficult  3: Somewhat easy  4: Very easy |
| Perceived behavioral control (PBC) | In the past 12 months, have you used a computer, smartphone, or other electronic means to look up medical test results? | Yes or No | 0: No  1: Yes |
| Seek Health Information (SHI) | Have you ever looked for information about health or medical topics from any source? | Yes or No | 0: No  1: Yes |
| Health Knowledge (HK) | Overall, how confident are you about your ability to take good care of your health? | 5: Not confident at all  4: A little confident  3: Somewhat confident  2: Very confident  1: Completely confident | 1: Not confident at all  2: A little confident  3: Somewhat confident  4: Very confident  5: Completely confident |
| Caregiving Status (CG) | Are you currently caring for or making health care decisions for someone with a medical, behavioral, disability, or other condition? | Yes or No | 0: No  1: Yes |
| Chronic Conditions (CC) | Diabetes or high blood sugar? | Yes or No | For each chronic condition, respondents answered ‘Yes’ (coded as 1) or ‘No’ (coded as 0). The extent of chronic conditions is the sum of the values. Varying from 0 to 6. |
|  | High blood pressure or hypertension? |  |  |
|  | A heart condition such as heart attack, angina, or congestive heart failure? |  |  |
|  | Chronic lung disease, asthma, emphysema, or chronic bronchitis? |  |  |
|  | Depression or anxiety disorder? |  |  |
|  | Have you ever been diagnosed as having cancer? |  |  |
| Preventive Health Behavior (PHB) | About how many cups of fruit (including 100% pure fruit juice) do you eat or drink each day? | 0 to 6 (None to 4 or more cups) | (eat three or more cups of fruit, eat four or more cups of vegetable, exercise 3 or more days per week)  3 if does all three 2 if does any two of three 1 if does any one of three |
|  | About how many cups of vegetables (including 100% pure vegetable juice) do you eat or drink each day? | 0 to 6 (None to 4 or more cups) |  |
|  | In a typical week, how many days do you do any physical activity or exercise of at least moderate intensity, such as brisk walking, bicycling at a regular pace, and swimming at a regular pace (do not include weightlifting)? | 0 to 7 (None to 4 7 days per week) |  |
| Issue Involvement (II) | In the past 12 months, not counting times you went to an emergency room, how many times did you go to a doctor, nurse, or other health professional to get care for yourself? | 0: None  1: 1 time  2: 2 times  3: 3 times  4: 4 times  5: 5-9 times  6: 10 or more times | 0: None  1: 1 time  2: 2 times  3: 3 times  4: 4 times  5: 5-9 times  6: 10 or more times |
| Enjoy time in Sun (ETS) | To what extent do you enjoy spending time in the sun? | 4: Not at all  3: A little  2: Some  1: A lot | 1: Not at all  2: A little  3: Some  4: A lot |
| Morning-night person (MNP) | Which do you consider yourself to be? | 1: I’m definitely a morning-person  2: I’m more of a morning-person than a night-person  3: I’m neither a morning-person nor a night-person  4: I’m more of a night-person than a morning-person  5: I’m definitely a night-person | 1: I’m definitely a morning-person  2: I’m more of a morning-person than a night-person  3: I’m neither a morning-person nor a night-person  4: I’m more of a night-person than a morning-person  5: I’m definitely a night-person |
| Male | Are you male or female? | Male or Female | 0: Female  1: Male |
| Age | What is your age? |  |  |
| Education | What is the highest grade or level of schooling you completed? |  | 0: Up to High school 1: More than High school |
| Race | Black |  | 0: Non-Black  1: Black |
|  | White |  | 0: Non-White  1: White |
|  | Other |  | 0: Black or White  1: Non-Black and Non-White |
| Scale: it represents how questions’ option were provided in the survey.  Coding: It represents how these options were used in this analysis. | | | |
